# Collaborative impact of bacterial exometabolites governing root microbiota formation

**DOI:** 10.1007/s44154-023-00121-1

**Published:** 2023-09-07

**Authors:** Hafiz Abdul Kareem, Xinwei Hao, Xihui Shen

**Affiliations:** State Key Laboratory of Crop Stress Biology for Arid Areas, Shaanxi Key Laboratory of Agricultural and Environmental Microbiology, College of Life Sciences, Northwest A&F University, Yangling, Shaanxi China

**Keywords:** *Pseudomonas*, Exometabolites, Root microbiota, Operons, Pyoverdine

## Abstract

The majority of the root microbiota formation derives from soil-dwelling microorganisms. The limited extent of thorough investigation leads to a dearth of knowledge concerning the intricate mechanisms of microbe-microbe interaction implicated in the establishment of root microbiota. Therefore, the taxonomic signatures in bacterial inhibition profiles were determined by in vitro testing of 39,204 binary interbacterial interactions. However, findings from genetic and metabolomic studies elucidated that co-functioning of the antimicrobial 2,4-d iacetylphloroglucinol (DAPG) and the iron chelator pyoverdine as exometabolites has significantly contributed to the potent inhibitory activities of the highly antagonistic Pseudomonas brassicacearum R401. Microbiota restoration with a core of *Arabidopsis thaliana* root commensals showed that these exometabolites possess a root niche-specific function in establishing root competence and inducing anticipated changes in root surroundings. Both biosynthetic operons are abundant in roots in natural habitats, indicating that these exometabolites co-functioning is an adaptive feature that helps *Pseudomonad* dominate the root microbiota.

Root microbiota, the community of bacteria underneath plant roots, is characterised by low taxonomic diversity and dominant taxonomic classes (Bai et al. 2015; Lundberg et al. 2012). Carbon-rich photo-assimilates are continually generated by plants in the rhizosphere and operate as significant mediators of root microbiota formation and activity, which are determined by host genetic factors (Eilers et al. 2010; Wippel et al. 2021). Root commensals may play a role in the microbiota’s formation via the production of specialized metabolites that may be involved in cooperative or competitive partnerships with different bacterial taxa. Producing specialized inhibitory metabolites (Clough et al. 2022; Fira et al. 2018) involves complex enzymatic mechanisms that are energetically costly. These mechanisms are carried out via biosynthetic gene clusters (BGCs) (Crits-Christoph et al. 2018). Antimicrobial metabolites like 2,4-diacetyl phloroglucinol (DAPG) produced by fluorescent Pseudomonas serve a significant role not only in preventing the spread of the fungal pathogen-mediated take-all disease but also in restricting bacterial growth (Zhou et al. 2014). In addition, the presence of growth-promoting siderophores can enhance the ability of bacterial root pathogens to cause infections. The pathogenicity of *Ralstonia solanacearum (Rs)* is attributed to its ability to sequester inorganic iron through the use of siderophores, which limits the availability of this essential nutrient to other microorganisms (Gu et al. 2020). However, it’s still unclear whether these exometabolites from bacteria with varied modes of action alter root microbiota assembly and enhance strain prevalence on roots. To address this prevailing conundrum, extensive research effort has been published by Getzke et al. (2023) in the well-known journal PNAS that aims to assess the prevalence of antagonistic binary interactions among the root microbiota by utilizing a group of commensal microorganisms derived from *Arabidopsis thaliana* roots (At-Sphere). Root microbiota formation was shown to be supported by the antibiotics DAPG and pyoverdine (the high-affinity iron chelator), both of which were found by Getzke and co-workers as exometabolites underlying the actions of the highly hostile *Pseudomonas brassicacearum* R401.

They examined the pervasiveness of interbacterial competition initiated by secreted metabolites by analysing 39,204 binary interbacterial interactions, comprising 198 producers and 198 target isolates. All tested bacterial classes exhibited antagonistic interactions, suggesting that the formation of exometabolites is ubiquitous around the At-Sphere culture database of commensals (Bai et al. 2015). This demonstrated that only a small number of bacteria, mainly from the family Pseudomonadaceae, showed extensive inhibition of isolates from different phyla. They found that root-derived bacteria had 2.7 times more inhibitory activity than soil-derived bacteria, implying that the exometabolite production may be beneficial for bacterial root colonization. *Ralstonia spp.* are the primary root pathogens of robust Arabidopsis plants in nature. Getzke et al. (2023) postulated that the root microbiota of *A. thaliana* of bacterial origin plays a pivotal role in averting diseases in its natural habitat. Furthermore, 167 bacterial strains were subjected to inhibitory assays against pathogenic *Rs*. A particular assemblage of bacteria originating from the root and soil exhibited growth-inhibitory effects against *Rs* in modified Burkholder plate-based assays (mBA). The dominant manifestation of inhibitory activities was observed by bacterial strains of three distinct genera: *Pseudomonas, Streptomyces,* and *Bacillus*.

The fundamental ground of Getzke et al. (2023) postulation was that the biosynthesis of specialized metabolites is mediated by genome-encoded potential, which is interrelated with the ability of inhibitory halo production. The results of the mBA experiments indicate that the predicted BGCs strains exhibited a significant enrichment of halo-producing isolates compared to nonproducers. This observation suggests these compounds may play a crucial role in interbacterial competition. Network analysis of mass spectra uncovered that Gammaproteobacteria comprise the most class-specific metabolites, signifying that their potent inhibitory activity may be attributed to the production of an extensive collection of specialized metabolites. Further, a complementary investigation was conducted to assess differences in the interaction between specialized metabolites and competitors using metabolomes derived from 16 inhibitory zones originating from 10 producer isolates that exhibit extensive inhibitory potency against three target isolates. The network's dereplication showed that interactions among different bacterial strains selectively stimulated polyketide production, suggesting a potential function for these substances in interbacterial competition. Inhibitory action against 50 isolates from all examined bacterial groups, except *Bacilli* and *Flavobacteria*, was seen in *P. brassicacearum* R401 due to its increased exometabolite synthesis. The antiSMASH technique for the R401 genome predicted 16 BGCs. Notably, a precise match of the *phl* operon of *Pseudomonas protegens Pf-5* was identified, leading to the inference that DAPG mediates the inhibitory activity of R401. Moreover, the outcome explicate that the R401 Δ*phlD* mutant retained 71% of its inhibitory action against *Rs* despite a significant reduction in its antagonistic activity. Therefore, it seems that the inhibitory action of R401 against *Rs* cannot be fully explained by DAPG alone (Fig. 1).

**Fig. 1:**
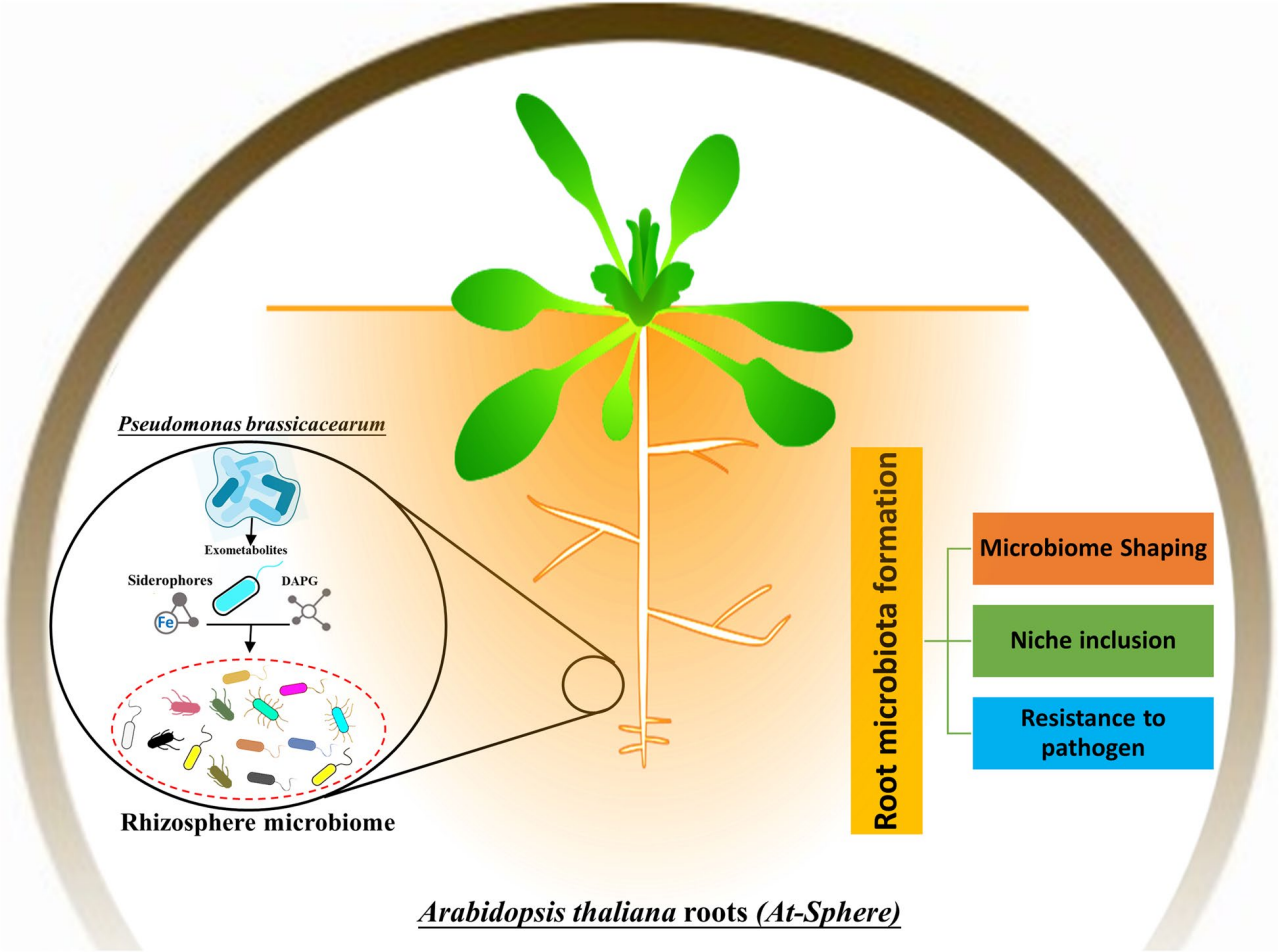
Graphical illustration of antimicrobial DAPG and the high-afffnity iron chelator pyoverdine as exometabolites underpinning the activities of the highly antagonistic *Pseudomonas brassicacearum *R401 for root microbiota establishment in *Arabidopsis thaliana*

A genetic screen was used to find out what was going on with the residual inhibitory effect of R401 *phld.* They constructed more than 6,000 insertion mutants using Tn5 transposon insertion technology and found mutant Δ*pvdy* and its downstream situated gene NRP synthetase Δ*pvdl* witnessed the contribution of R401 putative acyltransferase (*pvdY*) to the biosynthesis of pyoverdine and *Rs* inhibition in R401 (Lamont et al. 2006). Moreover, mass spectrometry findings elucidated that all generated pyoverdine mutants of R401 (Δ*pvdy* & Δ*pvdy*Δ*pvdl*) were incapable of producing pyoverdine. Getzke et al. (2023) postulated that pyoverdines help explain the remarkably high inhibitory activity of *Pseudomonadaceae* more generally; therefore, an additional mini-Tn5 mutant library of more than 6,000 *Pseudomonas* fluorescence R569 insertion mutants producing pyoverdines was generated. Unlike the R401, the inhibitory activity of *Rs* was entirely lacking in both R569 single mutants Δ*pvdy* and Δ*pvdl*. The acquired results suggest that pyoverdine could be one of the numerous exometabolites synthesized by root commensals that impede the growth of *Rs*. Thus, their iron chelator action is likely mediating their inhibitory effects against root commensal and pathogenic *Rs*. Moving forward, among all other tested isolates, the double DAPG and pyoverdine mutants (Δ*pvdy*Δ*phld* and Δ*pvdl*Δ*phld*) exhibited severely rebated inhibitory activity against *Rs* in comparison to the single mutants, justifying the concurrent influence of two metabolites. DAPG and pyoverdine jointly accounted for > 70% of the inhibitory activity of R401, but the presence of an inhibition residual halo pointing to another exometabolite has yet to be defined. The influence of DAPG and pyoverdine on the structure of root microbiota and *Rs* growth was studied by reconstitution experiments (Kremer et al. 2021). Unchanged shoot fresh weight with no wilting symptoms showed that *Rs* could not cause disease on *A. thaliana*, conceivably due to the existence of synthetic community (SynCom), justified by deficient root samples *Rs* relative abundances. Relative isolate abundances were inspected to determine physiological relevance in planta, and this study determined that the disruption of the associated BGCs in R401 only benefited DAPG- and/or pyoverdine-sensitive isolates. This particular observation has prompted the need to examine the impact on both community-level effects and individual isolates. The average decrease in inhibitory activity was observed across all R401 mutants. As a result, pairwise in vitro interaction experiments can correlate with the impact of two exometabolites produced by a single isolate on a taxonomically diverse SynCom's key ecological indices.

The relative abundance of R401 in SynCom samples from root and soil compartments determined the influence of R401-mediated bacterial assembly modulation and diversity on the competitiveness of R401. The findings indicated that DAPG and pyoverdine serve as root competence determinants of R401, particularly in competing with other SynCom members within the root compartment. Furthermore, the frequency of plant-associated *Pseudomonas* strains in natural soils was assessed to check their capability of producing DAPG and pyoverdine. The genomes of multiple commensal culture collections isolated from the roots or leaves of *A. thaliana* or the roots of the legume *Lotus japonicus* unleashed that root-derived *Pseudomonas* isolates had a greater abundance of DAPG and pyoverdine BGCs. The findings presented in this study corroborate the conclusions drawn from our obtained outcomes of specific gnotobiotic A. thaliana and core commensal communities and indicate a more comprehensive root-specific function of the two exometabolites in natural environments above the crucifer model. In summary, the integration of high-throughput binary interaction studies with genome mining for BGC collections of root microbiota can be used to detect strains that exhibit broad-spectrum antagonistic activities and are potentially potent root colonizers. This could have implications for future interventions using rational biologicals to intervene in the root microbiome in order to impart positive features on the host, such as indirect pathogen defence and better mineral nutrition.

## Data Availability

Not applicable.

## Abbreviations

BGCs: Biosynthetic gene clusters
DAPG: 2,4-Diacetylphloroglucinol
Rs: Ralstonia solanacearum
mBA: Modified Burkholder plate-based assays
SynCom: Existence synthetic community
